# Digital Mental Health Through an Intersectional Lens: A Narrative Review

**DOI:** 10.3390/healthcare14020211

**Published:** 2026-01-14

**Authors:** Rose Yesha, Max C. E. Orezzoli, Kimberly Sims, Aviv Y. Landau

**Affiliations:** 1 MedStar Health Research Institute, Columbia, MD 21044, USA; 2 Department of Medicine, Georgetown University Medical Center, Washington, DC 20057, USA; 3 Department of Social Sciences, Florida Memorial University, Miami Gardens, FL 33054, USA; max.orezzoli@fmuniv.edu; 4 Department of Psychiatry, Georgetown University School of Medicine, Washington, DC 20057, USA; kjs177@georgetown.edu; 5 Department of Psychiatry, MedStar Georgetown University Hospital, Washington, DC 20007, USA; 6 School of Social Policy and Practice, University of Pennsylvania, Philadelphia, PA 19104, USA; landauay@upenn.edu

**Keywords:** artificial intelligence, mental health, equity, marginalized communities, intersectionality, black, hispanic, LGBTQ+, neurodivergence

## Abstract

For individuals with mental illness who experience multidimensional marginalization, the risks of encountering discrimination and receiving inadequate care are compounded. Artificial intelligence (AI) systems have propelled the provision of mental healthcare through the creation of digital mental health applications (DMHAs). DMHAs can be trained to identify specific markers of distress and resilience by incorporating community knowledge in machine learning algorithms. However, DMHAs that use rule-based systems and large language models (LLMs) may generate algorithmic bias. At-risk populations face challenges in accessing culturally and linguistically competent care, often exacerbating existing inequities. Creating equitable solutions in digital mental health requires AI training models that adequately represent the complex realities of marginalized people. This narrative review analyzes the current literature on digital mental health through an intersectional framework. Using an intersectional framework considers the nuanced experiences of individuals whose identities lie at the intersection of multiple stigmatized social groups. By assessing the disproportionate mental health challenges faced by these individuals, we highlight several culturally responsive strategies to improve community outcomes. Culturally responsive strategies include digital mental health technologies that incorporate the lived experience of individuals with intersecting identities while reducing the incidence of bias, harm, and exclusion.

## 1. Introduction

The United States is suffering a profound mental health crisis. Finding accessible and effective care is paramount when a health condition is urgent, yet no organized system for crisis mental health care exists. The consequences are multifaceted: distress for people in crisis, overburdened law enforcement and hospital emergency departments, exponentially increasing rates of suicide, financial burden, and acts of violence by and upon individuals in mental distress [1,2]. The ubiquitous use of AI has allowed a global dialogue about the possible benefits and shortcomings of digital mental health. Moreover, there exists an increasing necessity to address the potential impact of widely enhanced, accessible, and conversational AI on mental health. Feelings of loneliness coupled with barriers to accessing mental health services have made LLM-based chatbots more attractive to users seeking support [3]. Globally, approximately 970 million people are living with a mental disorder. Mental disorders are increasingly recognized as a leading cause of disease burden [4]. The widespread incorporation of AI in daily use has catalyzed a global discussion about the possible benefits and risks of AI on mental health. There is an urgent need to investigate the influence of widely accessible AI on mental health [5]. AI-driven tools hold the potential for producing a positive impact on mental health. Algorithms can be used to assess data by linking lifestyle, genetic, and environmental markers to evaluate the best treatment option for every individual [6]. AI-enabled tools can help prevent more severe mental illness by identifying populations who exhibit high-risk markers [7]. AI also shows promise in reducing barriers to treatment and providing timely access to mental health care [8]. While AI shows promise in the early identification of risk and treating large populations of patients, significant weaknesses remain [9], including biases that may result in incorrect assessment. Trusting DMHAs requires highlighting their potential to personalize care and being transparent about safety implications.

DMHAs have rapidly gained recognition, promising innovative solutions and personalized interventions. Current efforts have focused on creating computational approaches to diagnosing and treating mental illnesses [10]. Additionally, digital mental health tools can deliver information and coping strategies to users instantaneously [11,12]. Text-based messaging with a human or with a machine (chatbots) has become pervasive in the past few years. AI conversational agents could potentially provide contextual and instantaneous support. *Woebot*, for example, treats “depression and anxiety using a digital version of time-tested cognitive behavior therapy” [13]. Large language models (LLMs) connected to frequently used chatbots (e.g., OpenAI’s GPT and Google’s Gemini) hold significant promise to support and even automate psychotherapy. Interest in such applications is rapidly growing in the mental health sector. However, due to the complex nature of mental illness, guardrails are needed to keep users safe [14]. In the *Mad in America* blog entitled “How Chatbots Deepen the Mental Health Crisis”, Kingsmith argues that AI chatbots use passive agreement to prioritize user engagement over safety. He further states that chatbots generally lack genuine empathy and users are transformed into recipients of generalized affirmations, void of the genuine empathy needed for human well-being [15].

While LLMs are not designed or intended for mental health support, preliminary evidence has found that millions of Americans with mental health conditions are turning to them for guidance, theoretically making these technologies one of the largest providers of mental health services in the United States [16]. However, LLMs may also exacerbate disparities via algorithmic bias. Algorithmic bias can originate from several sources, such as a lack of diverse representation in AI research and development teams, bias in training data, and failure to address the wider sociocultural framework [17,18,19]. Equity has become a key concern in the promise of digital platforms to transform healthcare [20,21]. Mental healthcare AI has the potential to reduce implicit bias in diagnosis and treatment, with pattern recognition techniques, personalized diagnostics, and clinical decision support systems, such as the use of AI scribes [8,22]. However, in practice, LLMs usually produce more errors when analyzing mental health data from marginalized groups [23]. It is likely that models trained on biased data may exacerbate existing inequities unless solution-focused measures are implemented [24]. LLMs may also highlight unsubstantiated claims about mental illness, increasing existing biases in psychiatric diagnosis and treatment in minority populations. LLMs may also learn and reproduce stigmatizing language located in electronic health records [25].

This paper examines how digital mental health interventions can improve health equity by highlighting the lived experience of individuals across multiple axes of marginalization, using an intersectional framework that considersimpact belonging to multiple stigmatized social groups has on the safety and effectiveness of digital mental health tools. Intersectional theory calls attention to the reality that individuals embody several identities simultaneously that can be vulnerable to discrimination, magnifying their risk of stigmatization [26]. Our narrative review synthesizes current evidence on the use of AI for mental health, evaluates its impact on marginalized communities through an intersectional lens, and identifies solution-based strategies to address biases and shortcomings of current technologies for digital mental health.

## 2. Methods

This narrative review was conducted using an intersectional framework [27] to examine the impact of digital mental health interventions on marginalized communities. Literature was selected through searches of PubMed, JSTOR, ACM Digital Library, MEDLINE, PsycINFO, arXiv, and Google Scholar, using combinations of terms such as “digital mental health,” “algorithmic bias”, “artificial intelligence, “large language models”, “racial bias”, “neurodivergence”, “intersectionality,” “equity,” and “marginalized populations.” Around 120 empirical sources published between 2006–2025 were used for this narrative review. The inclusion criteria focused on the impact of digital mental health on racial/ethnic minorities, LGBTQ+ individuals, and neurodivergent populations. We excluded studies that did not report basic methodological details on the impact of DMHAs on the mental well-being of individuals with intersecting identities. The majority of our sources include peer-reviewed publications. In addition, approximately 10 opinion pieces from major news outlets were integrated to ensure a diverse perspective, understand critical insights, and explore any knowledge gaps. These sources were included as contextual references in our review, rather than primary sources, and relied on non-systematic anecdotal evidence. Intersectional identities exist in many permutations that span multiple axes of discrimination (e.g., gender, race, ability, class, sexuality, neurodivergence). One limitation of our review is that not all of these combinations were explored; further research investigating a variety of experiences is needed.

## 3. Narrative Review

### 3.1. The Role of AI in Mental Health Care

The incidence of mental health diagnoses and psychotropic prescriptions has continued to rise in recent years [28,29,30]. Amidst this turmoil, many have found AI to be a potentially revolutionary tool. AI chatbots reduce barriers to mental health support, particularly for individuals with mild distress. Conversational AI that can convincingly mimic human speech may reduce demand on overburdened mental health systems [14,31]. Wysa, a Smartphone-Based AI Chatbot App for Mental Well-Being, was created as an AI-based chatbot app focused on increasing mental well-being by using a text-based platform. The app works by reacting to emotions that a user expresses in text conversations and utilizes evidence-based self-help tools such as CBT, dialectical behavior therapy, positive behavior support, and behavioral reinforcement, and mindfulness, to allow users to build emotional resilience skills [32]. A systematic review and meta-analysis synthesized evidence on the effectiveness of AI-based conversational agents (CAs) used for mental well-being. The findings of this review highlight that CAs may effectively alleviate mental distress, with the most significant effects seen in research using generative AI, using multimodal or voice-based CAs, or providing interventions with mobile applications and instant messaging platforms [33]. The use of AI for treatment and intervention in mental illness demonstrates a transformative shift in how we approach mental illness. The accessibility of digital mental health tools eliminates barriers to treatment that have historically prevented individuals from accessing treatment.

### 3.2. Bias in Digital Mental Health Diagnosis and Treatment

Digital mental health systems offer the opportunity to identify various psychological patterns, assist with triage, and support treatment plans that broaden access to care. Despite the transformative potential of DMHAs, AI-powered mental health tools may replicate biases propelled by structural inequities. Therefore, it is critical to examine how these biases affect the lived experiences of marginalized users. Many individuals face significant barriers to timely and effective mental healthcare. Several forms of bias can arise throughout the lifecycle of AI systems, including during data collection, clinical documentation, model design, and implementation, which perpetuate existing disparities in mental health care [34,35,36,37]. Careful attention to how inequities emerge across these stages is crucial before AI systems are implemented for widespread clinical use.

Historical disparities in psychiatric diagnosis and treatment underscore the risks. For example, historical disparities in psychiatric diagnosis and treatment have shown that Black patients have been disproportionately overdiagnosed with psychotic disorders, whereas Asian American and Hispanic patients have been underdiagnosed with mood disorders. In addition, Black patients also experience high rates of coercive interventions, such as compulsory hospitalization, and are frequently subjected to more aggressive forms of treatment by clinicians [38,39,40].

Comparable disparities are now also evident in digital contexts. Black communities are less likely to have mental health problems identified by digital mental health tools because AI lacks a critical understanding of how marginalized communities communicate and perceive different cultural contexts [41,42,43]. Several factors increase the incidence of bias in AI systems. For example, NLP tools may misinterpret African American English and different aspects of cultural representations of marginalized communities, resulting in misclassification and biased recommendations.

Furthermore, clinical documentation and electronic health records often contain implicit and explicit forms of bias concerning marginalized patients. When these data sets are used for training AI systems, they can reproduce disparities and promote bias in mental health diagnosis and treatment [23,25,44,45,46,47]. A lack of accuracy also occurs in speech-based machine learning models, particularly for Black women, as they can incorrectly diagnose anxiety and depression, prompting concerns about fairness and equity in AI-driven healthcare [48].

The implications of these biases extend beyond technical design and affect both clinical practice and health policy. Incorporating biased AI systems into healthcare can reinforce mistrust among marginalized communities, as these systems may perpetuate existing biases and inequities [49,50]. To reduce these biases in AI, it is necessary to go beyond algorithmic fairness by emphasizing patient-centered outcomes, which involves considering patients’ lived experiences and promoting inclusive design principles. This includes highlighting the significance of community-based participatory research and equity-centered design in the development of digital mental health interventions that are both clinically effective and socially responsive [21,51,52]. Without such safeguards, AI will likely reproduce rather than mitigate existing inequities, leaving marginalized populations with continued barriers to high-quality mental health care [53,54].

Ambient AI scribes, voice-to-text transcription tools that use LLMs to generate medical notes from recorded clinician-patient encounters, are poised to have a seismic impact on the delivery of mental healthcare, since they are being rapidly utilized in a wide variety of healthcare settings [55]. However, inadequate attention has been given to how their use may affect marginalized populations. An emerging body of literature identifies important ethical considerations for the deployment of ambient AI scribes in mental health care contexts, highlighting the increased risk of patient harm from compromised privacy and errors resulting from LLM-mediated transcription and diagnostic reasoning [56,57,58,59,60,61]. These risks are augmented for members of vulnerable populations, who already experience higher rates of negative outcomes such as misdiagnosis due to biased mental health documentation practices that LLMs are likely to amplify [62,63,64,65,66,67].

## 4. An Intersectional Framework

### 4.1. Intersectionality

The term “intersectionality” refers to the premise that race, class, age, gender, ethnicity, sexuality, ability, and nation do not work as singular, mutually exclusive entities, but rather as categories that interact to produce multifaceted social inequalities [68,69]. The concept of intersectionality also provides a framework for understanding how individuals can be simultaneously empowered and oppressed through the ways their social identities intersect [27]. Digital mental health tools are often implicitly or explicitly designed to serve a white, male, Western, cisgender, straight, neurotypical population. However, a revised approach that addresses only one category of difference (e.g., a chatbot targeted at female users) is unlikely to be equally effective for all women, and could even exacerbate harm. Using an intersectional lens to create, evaluate, and deploy digital mental health tools could help address inequalities by helping to identify the multifaceted, intricate needs that reflect many complex variations in lived experience. Intersectional approaches may be of particular significance in the mental health field, since patients are already more likely to suffer from stigma and discrimination [70]. Using AI mental health tools with an intersectional approach offers the ability to account for a fuller range of possible benefits and pitfalls for utilization by marginalized groups than has previously been offered [71]. This review examined how digital mental health tools may disproportionately harm individuals with intersecting marginalized identities by reinforcing diagnostic bias, excluding culturally specific expressions of distress, and heightening feelings of mistrust.

### 4.2. Racial/Ethnic Disparities

One of the greatest challenges in using diagnostic instruments for mental health is that tests developed for the general population do not always represent minorities accurately. Many examiners tend to over-pathologize the same item response elicited by minority members [72] compared to middle-class Caucasians on which tests are normed. The research has shown that individuals in racial and ethnic minority groups are 20–50% less likely to initiate mental health service use and 40–80% more likely to drop out of treatment prematurely [49,50]. The latest improvements in AI technology suggest that as AI improves, there is potential that it may quickly progress to identify risk for personalized interventions, particularly in minority populations. However, LLMs such as GPT-5 are developed with a predominantly Western perspective, which does not adequately address the diversity of user needs across various cultural or social backgrounds [73]. Historical and contemporary adverse events have bred mistrust of the medical and mental healthcare system, including the use of big data and AI applications, amongst the Black community, with biases, discrimination, and a lack of understanding of cultural sensitivities, which hinder seeking appropriate psychiatric care. For example, a study published in the Journal of Black Psychology elucidated young Black men’s experiences of abuse by mental health professionals and police officers, increasing feelings of mistrust [39].

From 2010–2019, Hispanic individuals were responsible for over half of the population growth in the United States [74]. There is a dire necessity for mental health care within Hispanic populations [75]. However, relative to adults in other racial-ethnic groups, Hispanic adults were found to be less inclined to seek treatment for mental distress [76]. Mental health services that integrate culture and language into clinical practice could result in an increased desire for treatment within Hispanic communities [77]. Digital mental health interventions (DMHIs) have shown promise in addressing these inequities; however, many are only available in English, creating barriers for non-English speakers. Research shows that mental health services in the individual’s native language promote better outcomes [78]. At present, there are a limited number of apps that have translation options or cultural adaptations [79]. Research has shown that compared to Wysa-English, Wysa-Spanish users logged more sessions and yielded more disclosures of distress [78]. Users also showed a preference for interventions with free text responses in Spanish, and Wysa-Spanish users logged more frequent terms associated with negative emotions, risk factors for self-harm and suicidal ideation. This highlights the critical need for platforms such as ChatGPT to adapt a more culturally inclusive interface.

### 4.3. Lesbian, Gay, Bisexual, Transgender, Queer, and/or Questioning (LGBTQ+)

The marginalization and lack of representation of LGBTQ+ individuals often hinder them from seeking mental health services, especially due to the risk of encountering therapists who do not adequately provide gender-affirming care. Chatbots focused on the LGBTQ+ population can give users a safe environment for sensitive conversations, enabling roleplay experiences like coming out and dating in a low-risk environment [80,81]. However, AI chatbots could also replicate harmful stereotypes as a result of biases in their training data. They may also inadequately address the multifaceted needs of LGBTQ+ individuals, which could contribute to feelings of isolation. In addition, these chatbots may offer advice that places users in vulnerable situations, such as coming out to family members who are not supportive [3].

LGBTQ+ individuals are increasingly utilizing chatbots for mental health support. The capabilities of LLM chatbots are most significant when contextualizing their influence on marginalized communities [3,82]. Individuals in the LGBTQ+ community are approximately three times more likely to suffer from anxiety, depression, and suicidal ideation [83,84,85]. Previous studies have found that a third of a geographically diverse sample of transgender youth in Canada attempted suicide in the last year [86]. These results imply a very grim reality for transgender youth. While chatbots have the potential to provide inclusive mental health resources for the LGBTQ+ community, biases in these technologies can exacerbate damaging stereotypes [3]. A benchmark study analyzing whether LLMS produces biases to the LGBTQ+ community [87] used a community-in-the-loop method to highlight explicit harms identified by the LGBTQ+ community. The results showed high WQ (WinoQueer) bias scores, which signified that homophobia and transphobia are significant problems in LLMs, and that anti-queer sentiment must be addressed.

LLMs may include many biases and stereotypes that cause extreme harm to the queer community. Models can exacerbate biases if human supervision is not present at every step of the training pipeline [87]. AI shows promise for the LGBTQ+ community; however, biases should be addressed to ensure that these technologies do not exacerbate stigma and discrimination. Inclusive models should be used to create AI systems that are fair and respectful of LGBTQ+ individuals [85]. It is important to note that existing AI and digital mental health evidence treats LGBTQ+ populations as a monolithic group, obscuring multiple axes of marginalization. For example, previous studies have shown that multiracial LGBTQ+ youth experienced the highest rates of race-based bullying [88]. Very limited research has examined how AI-driven mental health systems may differentially impact intersectional subgroups, such as Black transgender youth or autistic queer individuals, who may face increased risks of bias, misdiagnosis, and harm.

### 4.4. Neurodivergence

Neuronormativity is defined by elevating the status of neurotypical social behaviors as “normal”, thus introducing an imbalance of power between neurodivergent individuals, such as those diagnosed as being on the spectrum of autism [89]. Autistic individuals are frequently disadvantaged due to factors like stigma, discrimination, socioeconomic inequality, and poverty [90]. In a 2021 report, the United States National Institute of Health suggested that research shows that early diagnosis for autism is likely to have long-term positive effects on symptoms and development of skills [91,92,93,94,95]. Reducing barriers to services for autistic individuals is critical [96]. Neuronormativity has often resulted in dehumanizing comparisons to animals, robots, and other non-human entities, which increases the importance of safeguarding technological agents against replicating these stereotypes [97,98]. There is a growing interest in humanizing AI agents such as robots and chatbots. However, prior work in computing research has shown ways in which technologies may fail to highlight the needs of autistic and other neurodivergent (ND) individuals and increase their marginalization. Chatbots may evaluate legitimate struggles through a narrow, algorithmic process [15].

Another problem is the potential for LLMs to misunderstand or misrepresent the experiences of non-speaking neurodivergent individuals who use alternative communication methods. A 2024 study analyzed AI bias towards several neurodivergent conditions, including autism, schizophrenia, ADHD and OCD [99]. The authors discovered a profound level of bias towards terms related to autism and neurodiversity. Extremely high levels of bias were noted for tests related to violence, slurs, or obsessiveness. In addition, the research revealed negative sentiment for terms associated with autistic individuals with higher support needs. AI could contribute to personalized solutions by prioritizing specific needs of neurodivergent individuals [100]. While these apps show potential for neurodivergent individuals, the unique needs of the neurodivergent population should be considered, while also addressing the risks for perpetuating stigma or bias. Table 1. Includes a summary of findings for four AI mental health tools and resources for marginalized populations.

## 5. Culturally Responsive and Participatory Design for Inclusive AI

To safeguard against biases and inequities in healthcare systems, strategies are needed for diverse populations actively focusing on marginalized populations and leveraging data showing a wide range of patterns of behavior and conditions. An ontological database organization that mitigates bias risks through multiple data representations could facilitate the use of equitable LLMs in mental health care [102]. For example, mitigating the potentially damaging impacts of AI-driven mental health documentation will require a multi-faceted approach. Development of mental-health specific LLMs incorporating the diverse perspectives of potential beneficiaries, including clinicians and patients of different backgrounds, could increase the effectiveness of AI scribe tools and improve their cultural sensitivity and responsiveness [103,104,105,106]. More research testing the performance of existing ambient AI scribe tools with a variety of populations is needed to inform ethical decision-making about uptake and implementation [14,24,107,108,109]. Finally, teaching clinicians how to evaluate and modify the output of AI-scribes using a culturally sensitive lens and empowering patients to engage in self-advocacy in response to errors and stigmatizing language in the medical record will be a key component of harm reduction as we navigate this era of digital transformation [110,111,112,113,114]. Transforming AI through an intersectional lens must happen across multiple communities. Despite the democratization of digital technologies, identity markers remain vulnerable to AI systems [115]. Analyzing AI through an intersectional lens can help uncover detrimental power structures and create solutions that implement trustworthy and equitable AI systems.

## 6. Discussion

The proliferation of individuals using AI for mental health support has posed unique challenges. Digital mental health initiatives may fill a critical role in a system that is severely lacking in resources, funding, and accessibility. However, consequences for marginalized communities exist and should be addressed. An intersectional framework can be used to discover biases in existing AI and propose solutions for marginalized communities [115]. Our narrative review assessed the current literature on this topic while providing an intersectional lens for digital mental health. While our review was both iterative and interpretative, there were limitations to our approach.

This review did not include empirical validation and therefore its conclusions are conceptual. Additionally, the evolving nature of technological advancement has resulted in many of the included studies becoming outdated relatively quickly. Finally, although intersectionality produces a critical outlook, structural nuances also produce inherent limitations. It is a methodological challenge to account for the inclusion of race, gender, sexuality, and neurodivergence within AI-driven systems. Many intersectional issues may be underrepresented in this review. Additionally, most cited studies examine single-axis identities (e.g., race, sexual orientation, or neurodivergence), which limits the ability to draw accurate conclusions about intersectional experiences. These limitations can be addressed by future research initiatives that highlight real-world data, community-based participatory methods, and intersectional frameworks. For example, indicators of “poor–rich” and “gay–straight” highlight that AI algorithms have classified correlations between neurodivergent individuals and LGBTQ+ identity as well as socioeconomic inequity. Future research investigating AI bias against LGBTQ+ neurodivergent individuals and other multiply marginalized neurodivergent individuals would aid in debiasing efforts [99]. The engagement of marginalized communities in the co-creation of AI tools is critical to ensuring that digital mental health technologies promote equity.

## 7. Conclusions

AI in mental health has paved the way towards promising solutions and interventions. However, LLMs may reveal unfounded assumptions regarding mental health, perpetuating biases in psychiatric diagnoses and treatment in minority populations. This narrative review highlights the current literature on digital mental health using an intersectional lens by assessing the diverse needs of marginalized populations. We conclude our review with recent findings on comprehensive strategies that involve engaging marginalized communities in the creation of AI tools.

## Figures and Tables

**Table 1:** AI Mental Health Tools and Resources for Marginalized Populations.

AI Mental Health App	Population	Features	Findings
*Wysa-Spanish*	Hispanic	Mental wellness AI-chatbot in Spanish, utilizing cognitive behavioral therapy (CBT) and dialectical behavior therapy (DBT).	Users of Wysa-Spanish logged more sessions and had a higher volume for disclosures of distress. The findings emphasize the significance of linguistic and cultural variations in the development of DMHAs [78].
*Headspace Health*	BIPOC	A guided meditation app and support app for BIPOC users experiencing racism. Offers CBT modalities such as mindfulness and reframing exercises	Many mental health apps depend on BIPOC to facilitate programs that provide lived experience narratives, rather than creating their own platforms to address inequalities [101].
*WinoQueer*	LGBTQ+	Community-in-the-Loop Benchmark for Anti-LGBTQ+ Bias in Large Language Models	A benchmark study was conducted to assess whether large language models (LLMs) revealed high WinoQueer bias scores, highlighting that homophobia and transphobia are profound problems in LLMs [87].
*Hazel*	Autism	Hazel allows neurodivergent users access to a series of tests where AI interprets results and provides personalized strategies.	AI is used in Hazel to assess an individual’s behaviors and responses to determine which tools are most effective for the individual. AI could contribute to personalized solutions by prioritizing specific needs of neurodivergent individuals [100].

Table 1: Four AI Mental Health Tools and Resources (Wysa-Spanish, Headspace Health, WinoQueer & Hazel) are summarized in this table. These applications utilize artificial intelligence to provide a range of tools and services focused on improving mental health for Hispanic, BIPOC, LGBTQ+, and neurodivergent individuals.

## Data Availability

No new data were created or analyzed in this study. Data sharing is not applicable to this article.
